# Pilot RNA‐seq data from 24 species of vascular plants at Harvard Forest

**DOI:** 10.1002/aps3.11409

**Published:** 2021-02-14

**Authors:** Hannah E. Marx, Stacy A. Jorgensen, Eldridge Wisely, Zheng Li, Katrina M. Dlugosch, Michael S. Barker

**Affiliations:** ^1^ Department of Ecology and Evolutionary Biology University of Arizona Tucson Arizona 85721 USA; ^2^ Department of Ecology and Evolutionary Biology University of Michigan Ann Arbor Michigan 48109‐1048 USA; ^3^ Genetics Graduate Interdisciplinary Program University of Arizona Tucson Arizona 85721 USA

**Keywords:** community transcriptomics, NEON, polyploidy, RNA‐seq, transcriptome assembly

## Abstract

**Premise:**

Large‐scale projects such as the National Ecological Observatory Network (NEON) collect ecological data on entire biomes to track climate change. NEON provides an opportunity to launch community transcriptomic projects that ask integrative questions in ecology and evolution. We conducted a pilot study to investigate the challenges of collecting RNA‐seq data from diverse plant communities.

**Methods:**

We generated >650 Gbp of RNA‐seq for 24 vascular plant species representing 12 genera and nine families at the Harvard Forest NEON site. Each species was sampled twice in 2016 (July and August). We assessed transcriptome quality and content with TransRate, BUSCO, and Gene Ontology annotations.

**Results:**

Only modest differences in assembly quality were observed across multiple *k*‐mers. On average, transcriptomes contained hits to >70% of loci in the BUSCO database. We found no significant difference in the number of assembled and annotated transcripts between diploid and polyploid transcriptomes.

**Discussion:**

We provide new RNA‐seq data sets for 24 species of vascular plants in Harvard Forest. Challenges associated with this type of study included recovery of high‐quality RNA from diverse species and access to NEON sites for genomic sampling. Overcoming these challenges offers opportunities for large‐scale studies at the intersection of ecology and genomics.

Many questions in ecology and evolutionary biology increasingly require combining data from these fields at large scales. In particular, integrated, large‐scale analyses of multispecies ecological and phylogenetic data sets have become critical to understanding plant distributions and responses to climate change (Zanne et al., 2014; Swenson and Jones, 2017; Maitner et al., 2018; Enquist et al., 2019; McFadden et al., 2019; Rice et al., 2019; Baniaga et al., 2020; Gallagher et al., 2020; Román‐Palacios and Wiens, 2020). Recognizing this need, the National Science Foundation (NSF) recently launched the National Ecological Observatory Network (NEON) to generate large‐scale data in areas including species occurrence, phenology, and climate, for ecological communities across the United States (Collinge, 2018; Knapp and Collins, 2019). Metagenomic and genomic sampling are also being used to identify and estimate changes in abundance and composition of some taxa, especially microbial communities (https://www.neonscience.org/data). Although these data and analyses will be crucial for understanding ecosystem‐scale processes, the collection of genomic data from a broader array of species across NEON sites would allow researchers to further integrate ecological and evolutionary processes in the analyses of communities.

Genomic analyses of single species, although important, do not capture the larger patterns occurring within an interacting community of plants. Transcriptome profiling or genome sequencing of multiple species and individuals within a community will open new, integrative avenues of analysis and allow us to address existing questions that require sampling of floras and communities (Bragg et al., 2015; Fitzpatrick and Keller, 2015; Bowsher et al., 2017; Han et al., 2017; Swenson and Jones, 2017; Zambrano et al., 2017; Matthews et al., 2018; Subrahmaniam et al., 2018; Breed et al., 2019). This is especially true for understanding responses to climate change where community‐level analyses are needed to capture the interacting dynamics of different species responses (Liu et al., 2018; Komatsu et al., 2019; Snell et al., 2019). The integration of community‐level genomic data from non‐model species with ecological and trait data will improve our understanding of plant responses to climate change. Collecting genomic data at the community level with repeated sampling that mirrors other trait data collection will permit assessments of the genetic diversity of entire plant communities and how they change over time, estimates of gene flow and hybridization, measurement of in situ gene expression variation across species in response to shared climate events, and a genomic perspective on functional diversity within and between plant communities. Metagenomics analyses of microbiomes have transformed our understanding of and approaches for studying microbial biology (Fierer et al., 2012a, 2012b; Turner et al., 2013; Delgado‐Baquerizo et al., 2018; Jansson and Hofmockel, 2020). Similar plant community transcriptomics and genomics studies could open new avenues of research and provide the crucial data to understand plant responses to climate change.

To explore the potential and challenges of plant community transcriptomics, we conducted a pilot RNA‐seq study at the Harvard Forest NEON site. Whereas many RNA‐seq studies are focused on collections of related species, an approach that simplifies collection and RNA extraction, a major challenge of community‐level transcriptomics is that a diverse range of plant species need to be sampled for RNA extraction in the field. In this pilot study, we evaluated RNA‐seq results generated following a protocol that we developed (Field Setup 2 of Yang et al., 2017) for collecting material at distant field sites and returning samples by shipping. Harvard Forest was selected for this pilot study because of access to a field station that simplified the logistics of working with liquid nitrogen. At Harvard Forest, we sampled 24 species of vascular plants from sites adjacent to the NEON plot. Each species was sampled on two different dates one month apart (in July and August 2016), as close to the same time of day as possible. Species were selected from a phylogenetically diverse range of plants that included ferns, trees, and herbaceous annuals. These plants were selected because they represented the diversity of form and habit that is present in the deciduous forest community at Harvard Forest. Another potential challenge for plant transcriptomics is the abundance of polyploid species and cytotypes (Barker et al., 2016a). With potentially twice as many (or more) genes in a polyploid genome, these species could require more sequencing reads than related diploids to obtain reference transcriptomes of similar quality. To explore the impacts of polyploidy on transcriptome surveys, we made an effort to select sets of related polyploid and diploid species. Here, we give an overview of our data collection, present new reference transcriptomes and translated protein collections for each species, and evaluate the quality of these assemblies using multiple approaches.

## METHODS

### Taxon selection and sampling

The *Harvard Forest Flora* (Jenkins et al., 2008) was used to guide our taxonomic selections and find species to represent each category (diploid/polyploid). Putative diploids and neo‐polyploid species were identified from chromosome counts obtained from the Chromosome Counts Database (Rice et al., 2015). Congeneric species pairs were selected based on their phylogenetic relatedness. Our sampling included nine polyploid and 11 diploid species (Table 1). We could not determine the ploidal level of four species. The Harvard Forest Flora Database (Jenkins et al., 2008) was used to locate sampling sites.

**TABLE 1:** Summary statistics for RNA‐seq data sets, assemblies with a *k*‐mer = 57, and translations.

Species	Ploidy^a^	Chromosome no.	July SRA	Aug SRA	Total Gbp (July + Aug)	No. of scaffolds	Mean scaffold length (bp)	Scaffold N50 (bp)	No. of translated proteins	Mean translated length (nucleic acids, bp)	N50 (trans. nucleic acids, bp)
*Dryopteris carthusiana*	P	82	SAMN08277176	SAMN08277204	26	643,129	264.5	710	24,851	473.8	543
*Dryopteris intermedia*	D	41	SAMN08277187	SAMN08277216	22	529,510	267.1	822	22,595	587.1	702
*Dryopteris marginalis*	D	41	SAMN08277188	SAMN08277217	21	550,548	260.1	917	34,121	633.9	789
*Galium mollugo*	D	11	SAMN08277173	SAMN08277201	40	608,764	179.2	1028	25,040	692.9	822
*Galium tinctorium*	D	12	SAMN08277181	SAMN08277210	32	78,487	400.6	1091	16,610	811.2	1023
*Galium triflorum*	P	33	SAMN08277189	SAMN08277219	37	574,562	246	899	38,650	664.9	798
*Hypericum perforatum*	P	16	SAMN08277171	SAMN08277199	25	335,837	233.4	867	34,670	698.6	858
*Juglans cinerea*	D	16	SAMN08277174	SAMN08277202	18.2	569,859	359.5	1151	66,595	712.4	918
*Lonicera tatarica* var. *morrowii*	NA	9	SAMN08277167	SAMN08277195	10.4	386,927	324.9	1216	28,147	710.9	864
*Lysimachia ciliata*	P	48	SAMN08277190	SAMN08277220	24	1,422,451	207	828	32,005	631.6	777
*Lysimachia nummularia*	D	17	SAMN08277194	SAMN08277223	25.3	428,232	320.9	1102	46,343	716.7	894
*Lysimachia quadrifolia*	P	42	SAMN08277183	SAMN08277212	30	340,491	290.4	944	37,737	674.9	813
*Persicaria arifolia*	NA	NA	SAMN08277180	SAMN08277209	30	528,292	245	787	28,741	558.8	639
*Persicaria hydropiperoides*	NA	NA	SAMN08277172	SAMN08277200	21.3	347,558	344	913	46,058	658.8	795
*Persicaria sagittata*	NA	20	SAMN08277179	SAMN08277208	26	398,304	319.6	1148	33,118	725.1	885
*Plantago lanceolata*	D	6	SAMN08277178	SAMN08277206	31	213,834	293.1	1073	20,470	742.7	906
*Plantago major*	D	6	SAMN08277169	SAMN08277197	23	217,041	308.6	1032	24,715	782.6	993
*Plantago rugelii*	P	12	SAMN08277170	SAMN08277198	30	378,418	276.1	1161	30,673	760.5	930
*Polygonum cilinode*	D	11	SAMN08277184	SAMN08277213	17.3	1,065,186	186.8	415	6088	471.6	498
*Potentilla argentea*	P	21	SAMN08277177	SAMN08277207	29	245,734	425.6	1268	16,306	525.5	687
*Potentilla canadensis*	D	14	SAMN08277192	SAMN08277222	16.6	433,249	216.5	667	37,503	526.5	594
*Prunus serotina*	P	16	SAMN08277186	SAMN08277215	31	350,572	267.5	1017	30,812	658.3	774
*Prunus virginiana*	D	8	SAMN08277185	SAMN08277214	38	536,216	235	1110	38,773	678.8	813
*Reynoutria japonica*	P	33	SAMN08277193	SAMN08277224	44	410,810	279.6	870	34,662	549.3	609

Note

NA = not available; SRA = Sequence Read Archive.

^a^ P and D are polyploid and diploid species, respectively.

**FIGURE 1:**
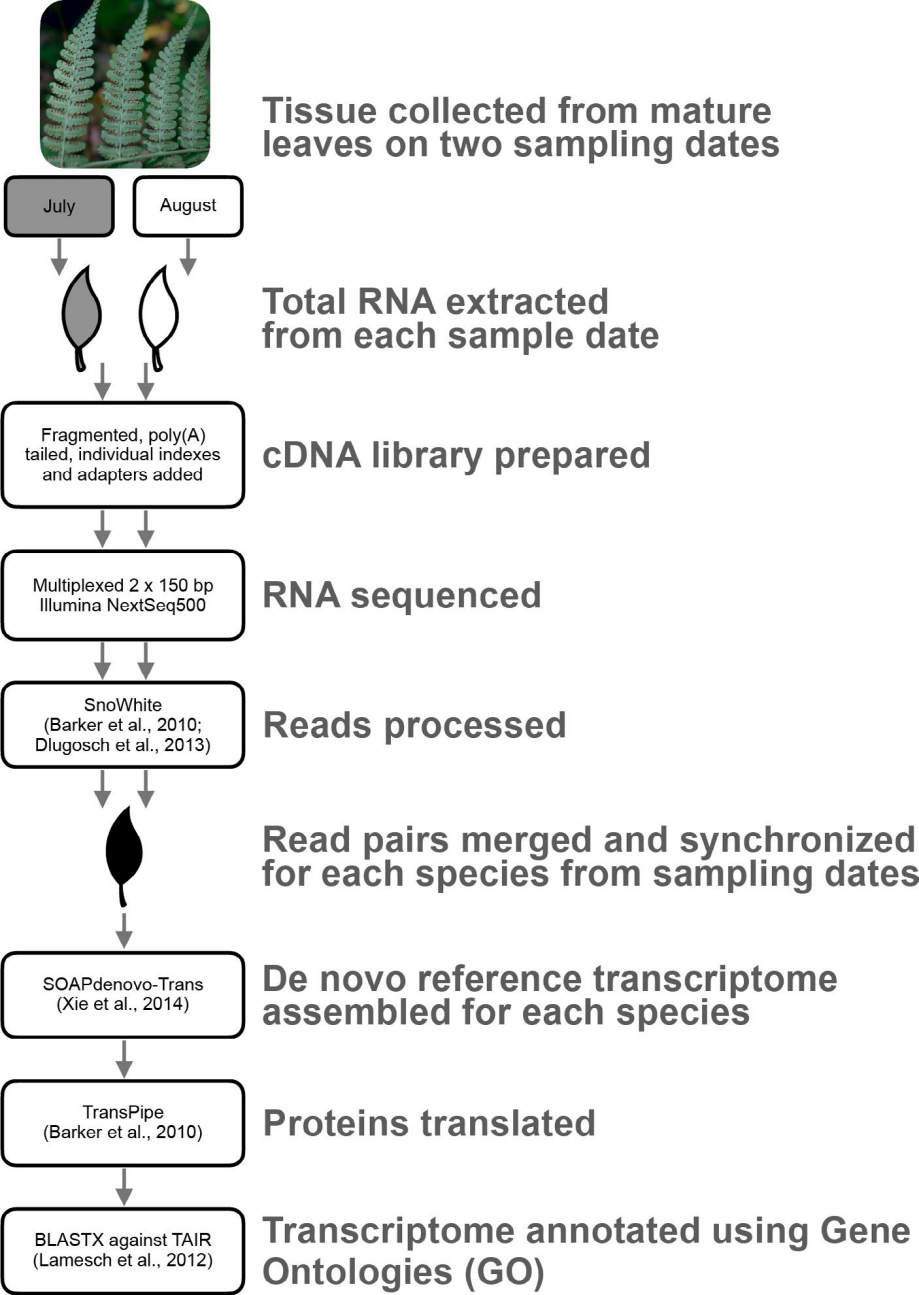
Workflow of our RNA‐seq collection and assembly.

Field collection for plant RNA‐seq followed the approach described in Yang et al. (2017). The only difference was here we sampled tissue from mature leaves of an apparently healthy individual (e.g., lacking herbivore or pathogen damage) rather than young flower or leaf buds to maintain developmental consistency as much as possible over time. Each target species was sampled from the same population on two different dates about one month apart (July and August) during the 2016 growing season (Fig. 1). We attempted to sample as close to the same time of day as possible on both dates by sampling species in the same order on both trips, but this was not always achievable due to challenges of fieldwork, such as weather and time to relocate sample populations. Leaf tissues were flash‐frozen in liquid nitrogen in the field and shipped on dry ice to the University of Arizona for RNA extraction. After leaf tissue collection, additional leaf tissue was preserved on silica for DNA backup, and the remaining plant material was pressed for a herbarium specimen (see Appendix 1 for voucher information and collection details).

### RNA extraction and RNA‐seq

Total RNA was extracted from leaf tissue collected on each sampling date for all species using the Spectrum Plant Total RNA Kit (Sigma‐Aldrich Co., St. Louis, Missouri, USA) following the manufacturer’s Protocol A. RNA was used to prepare cDNA using the Ovation RNA‐Seq System (catalogue no. 7102‐A01; NuGEN, Redwood, California, USA) via single primer isothermal amplification and automated on the Apollo 324 liquid handler (TaKaRa Bio, Kusatsu, Shiga, Japan). cDNA was quantified on the NanoDrop (Thermo Fisher Scientific, Waltham, Massachusetts, USA) and was sheared to approximately 300‐bp fragments using the Covaris M220 ultrasonicator (Covaris, Woburn, Massachusetts, USA). Libraries were generated using Kapa Biosystem’s library preparation kit for Illumina (KK8201; Roche, Wilmington, Massachusetts, USA). Fragments were end‐repaired and A‐tailed, and individual indexes and adapters (catalogue no. 520999; Bioo Scientific, Austin, Texas, USA) were ligated on each separate sample. The adapter‐ligated molecules were cleaned using AMPure beads (A63883; Agencourt Bioscience/Beckman Coulter, Indianapolis, Indiana, USA) and amplified with the KAPA HiFi enzyme (KK2502; Roche). Each library was then analyzed for fragment size on an Agilent TapeStation 4200 (Agilent Technologies, Santa Clara, California, USA) and quantified by qPCR (KAPA Library Quantification Kit, KK4835) on the QuantStudio 5 Real‐Time PCR System (Thermo Fisher Scientific). Multiplex pooling (13–16 samples per lane) and paired‐end sequencing at 2 × 150 bp were then performed on the Illumina NextSeq500 platform at Arizona State University’s CLAS Genomics Core facility. Raw read quality was assessed using FastQC (Andrews, 2010).

### De novo transcriptome assembly, protein translation, and quality assessment

Raw sequence reads were processed using the SnoWhite pipeline (Barker et al., 2010; Dlugosch et al., 2013), which included trimming adapter sequences and bases with a quality score below 20 from the 3′ ends of all reads, removing reads that are entirely primer and/or adapter fragments using TagDust (Lassmann et al., 2009), and removing poly(A/T) tails with SeqClean (https://sourceforge.net/projects/seqclean/). The cleaned reads from each sampling date were merged and cleaned to synchronize read pairs using fastq‐pair (Edwards and Edwards, 2019) and pooled to assemble a reference de novo transcriptome for each species.

Due to the significant time involved in running and evaluating multiple assemblies for each species, we chose five species that represent the phylogenetic diversity of our samples (*Dryopteris intermedia* (Muhl. ex Willd.) A. Gray, *Galium mollugo* L., *Juglans cinerea* L., *Plantago major* L., and *Persicaria sagittata* (L.) H. Gross) to identify the optimal *k*‐mer to use for assembling all 24 species. For these five exemplar taxa, we examined the quality of assemblies generated by SOAPdenovo‐Trans version 1.03 (Xie et al., 2014) across a range of *k*‐mers (37, 47, 57, 67, 77, 87, 97, 107, 117, and 127). Assembly quality across the different *k*‐mers was assessed by mapping the raw reads to each assembly with TransRate version 1.0.3 (Smith‐Unna et al., 2016) and evaluating the optimal assembly scores. TransRate calculates assembly scores by remapping the reads back to the assembly and combining a variety of metrics for each contig, including estimates of whether a base pair was called correctly, whether a base should be a part of the final transcript, the probability that a contig was derived from a single transcript, and the probability that a contig is structurally complete. We selected a *k*‐mer that produced the average highest optimal assembly score across the five species. This *k*‐mer (57, see Results) was used to assemble reference transcriptomes for the entire collection of species.

We used TransPipe (Barker et al., 2010) to identify plant proteins from the assembled transcripts for each reference transcriptome and provide protein and in‐frame nucleic acid sequences for each species. The reading frame and protein translation for each sequence was identified by comparison to protein sequences from 25 sequenced and annotated plant genomes from Phytozome (Goodstein et al., 2012). Using BLASTX (Wheeler et al., 2008), best‐hit proteins were paired with each transcript at a minimum cutoff of 30% sequence similarity over at least 150 sites. Transcripts that did not have a best‐hit protein at this level were removed. To determine the reading frame and generate estimated amino acid sequences, each transcript was aligned against its best‐hit protein by GeneWise 2.2.2 (Birney et al., 2004). Based on the highest‐scoring GeneWise DNA–protein alignments, stop and “N”‐containing codons were removed to produce estimated amino acid sequences for each transcript. Output included translated protein sequences and their corresponding nucleic acid sequences.

To assess the quality of the assembled transcriptomes for the full set of 24 species, we analyzed each with TransRate and BUSCO. Summary statistics, including the number of scaffolds, mean scaffold lengths, and N50, were calculated by TransRate version 1.0.3 for all scaffolds as well as for the subset of sequences that were identified as plant proteins and translated. We evaluated the completeness of our transcriptome coverage with BUSCO version 4.0.5 (Seppey et al., 2019). BUSCO compares sequences to a collection of universal single‐copy orthologs for the Viridiplantae (Viridiplantae Odb10) and the eukaryotes (Eukaryote Odb10). We also used the TransRate and BUSCO statistics to compare differences in the assemblies of diploid and polyploid species.

### Gene Ontology annotation and comparison

Gene Ontology (GO) annotations of all transcriptomes were obtained through translated BLAST (BLASTX) searches against the annotated *Arabidopsis thaliana* (L.) Heynh. protein database from TAIR (Lamesch et al., 2012) to find the best hit with a length of at least 100 bp and an *E‐*value of at least 1e‐10. GO‐slim annotations based on the plant GO‐slims from TAIR were obtained for the whole transcriptome for each species and presented as a heatmap. The heatmap columns were clustered by hierarchical clustering with default parameters in R with the order of GO categories set arbitrarily by the ranking in *Lysimachia ciliata* L. Rankings of the GO slim categories were determined by the relative frequency of the GO term among the transcripts in each transcriptome.

## RESULTS

**FIGURE 2:**
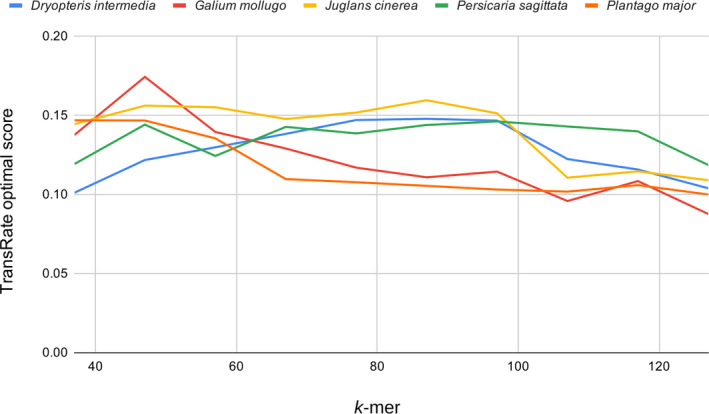
TransRate optimal scores for assemblies of five exemplar species from Harvard Forest. A reference transcriptome for each species was assembled with different *k*‐mers starting at *k* = 37 and increasing in increments of 10 to *k* = 127.

We found relatively little variation in the optimal TransRate scores across assemblies with different *k*‐mers. The optimal TransRate scores ranged from ~0.1–0.15, with each of the five exemplar species peaking at different *k*‐mers (Fig. 2). Scores trended downward for all species at higher *k*‐mers, with no sharp peaks in the score apparent in most taxa. The mean *k*‐mer of the top‐scoring assemblies for each species was 61, and the closest *k*‐mer to this value (57) was used to assemble reference transcriptomes for all 24 species.

**FIGURE 3:**
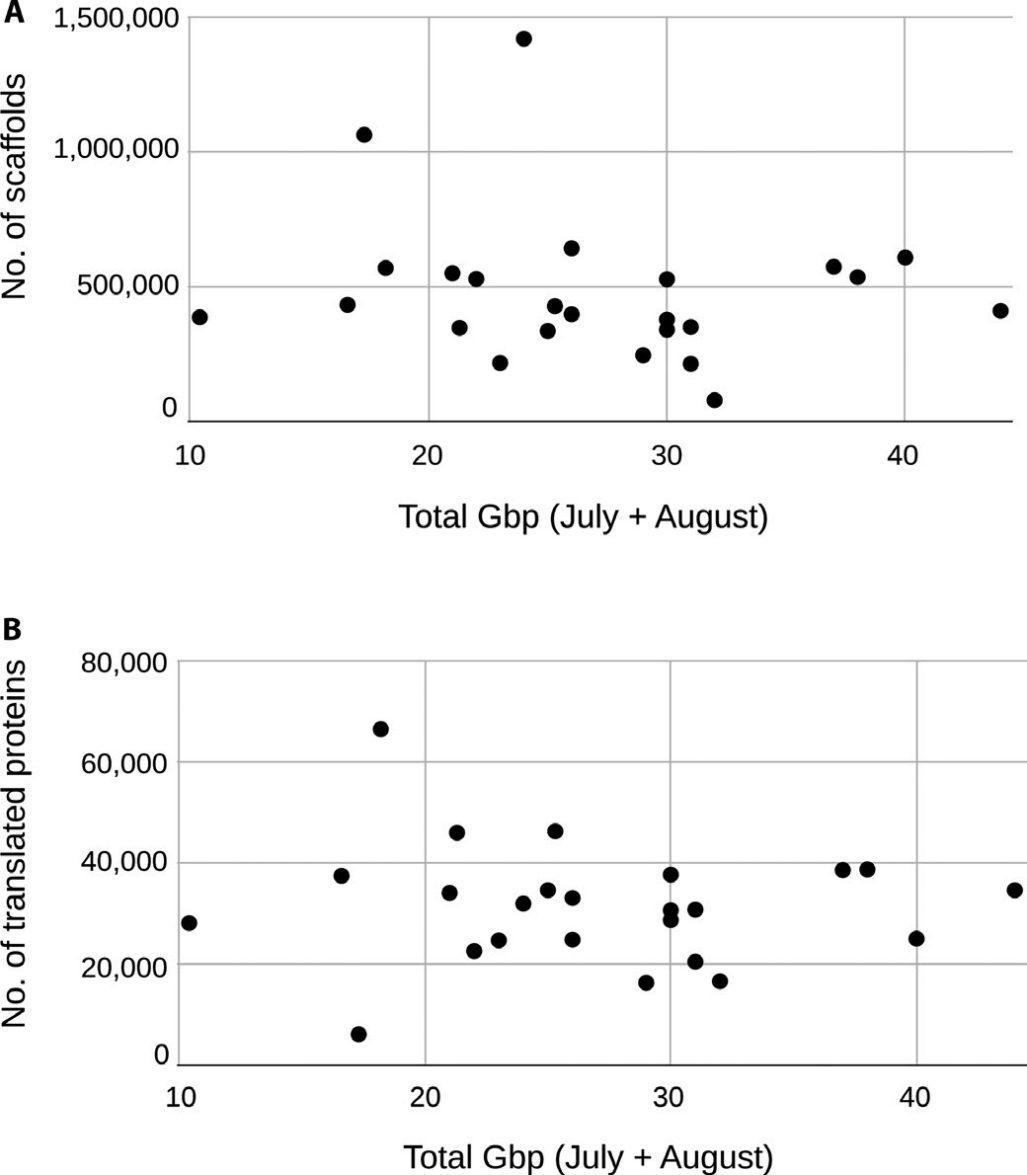
Comparison of the number of (A) scaffolds and (B) translated proteins produced by each assembly with the total number of giga base pairs (Gbp) sequenced for each species.

**FIGURE 4:**
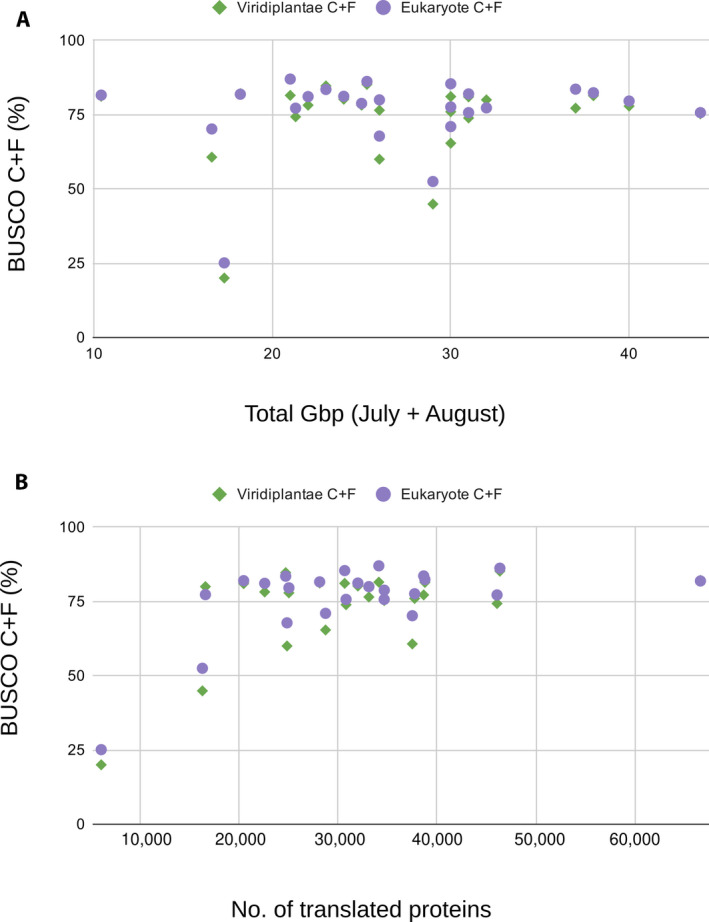
The percentage of BUSCO complete (C) plus fragmented (F) matches compared to the (A) total giga base pairs (Gbp) sequenced and (B) number of translated proteins in each assembly. Green diamonds represent BUSCO matches to the Viridiplantae database, whereas purple circles represent matches to the eukaryote database.

Assemblies for most of the 24 species appeared to be of relatively high quality. By combining RNA‐seq libraries representing two different time points, we obtained an average of 27 Gbp of reads for each reference transcriptome (Table 1). With a *k*‐mer = 57, the assemblies contained an average of 483,084 scaffolds with a mean length of 281 bp and N50 of 960 bp. The translated nucleic acids for each assembly had an average of 31,470 sequences with a mean length of 652 bp and N50 of 789 bp. We observed no significant relationship between the number of scaffolds or number of translated proteins and sequencing depth (Fig. 3). The mean complete plus fragmented BUSCO percentages were 73.2% against the Viridiplantae database and 76% against the eukaryote database (Table 2). We found that the number of hits to sequences in the BUSCO databases plateaued at around 20 Gbp of sequencing effort and around 20,000 proteins (Fig. 4).

**TABLE 2:** BUSCO results for comparisons to the Viridiplantae and eukaryote databases.

Species	BUSCO Viridiplantae database							BUSCO Eukaryote database						
	C	(S)	(D)	F	M	D/S	C+F	C	(S)	(D)	F	M	D/S	C+F
*Dryopteris carthusiana*	19.3	16.9	2.4	40.7	40	0.142	60	24.3	20	4.3	43.5	32.2	0.215	67.8
*Dryopteris intermedia*	39.8	35.1	4.7	38.4	21.8	0.134	78.2	48.2	44.3	3.9	32.9	18.9	0.088	81.1
*Dryopteris marginalis*	41.5	34.4	7.1	40	18.5	0.206	81.5	48.6	43.5	5.1	38.4	13	0.117	87
*Galium mollugo*	27.8	22.6	5.2	50.1	22.1	0.230	77.9	38	32.5	5.5	41.6	20.4	0.169	79.6
*Galium tinctorium*	40	37.9	2.1	40	20	0.055	80	50.2	46.3	3.9	27.1	22.7	0.084	77.3
*Galium triflorum*	30.8	21.9	8.9	46.4	22.8	0.406	77.2	33.4	21.6	11.8	50.2	16.4	0.546	83.6
*Hypericum perforatum*	29.5	22.4	7.1	48.7	21.8	0.317	78.2	38.8	29	9.8	40	21.2	0.338	78.8
*Juglans cinerea*	33.9	27.8	6.1	48.2	17.9	0.219	82.1	47.8	39.2	8.6	34.1	18.1	0.219	81.9
*Lonicera tatarica* var. *morrowii*	34.8	29.4	5.4	46.4	18.8	0.184	81.2	47.1	36.9	10.2	34.5	18.4	0.276	81.6
*Lysimachia ciliata*	34.1	28.7	5.4	46.1	19.8	0.188	80.2	49	40	9	32.2	18.8	0.225	81.2
*Lysimachia nummularia*	42.4	35.1	7.3	42.8	14.8	0.208	85.2	48.6	34.9	13.7	37.6	13.8	0.393	86.2
*Lysimachia quadrifolia*	31.8	26.4	5.4	44.2	24	0.205	76	38	32.5	5.5	39.6	22.4	0.169	77.6
*Persicaria arifolia*	13.9	10.6	3.3	51.5	34.6	0.311	65.4	24.7	16.9	7.8	46.3	29	0.462	71
*Persicaria hydropiperoides*	25.4	16.5	8.9	48.9	25.7	0.539	74.3	32.9	18.4	14.5	44.3	22.8	0.788	77.2
*Persicaria sagittata*	27.8	16.5	11.3	48.7	23.5	0.685	76.5	39.2	20.4	18.8	40.8	20	0.922	80
*Plantago lanceolata*	40.5	36	4.5	40.5	19	0.125	81	45.9	39.2	6.7	36.1	18	0.171	82
*Plantago major*	51.3	48	3.3	33.4	15.3	0.069	84.7	54.1	49.8	4.3	29.4	16.5	0.086	83.5
*Plantago rugelii*	34.5	20.9	13.6	46.6	18.9	0.651	81.1	45.8	27.8	18	39.6	14.6	0.647	85.4
*Polygonum cilinode*	11.5	9.4	2.1	8.5	80	0.223	20	5.5	4.7	0.8	19.6	74.9	0.170	25.1
*Potentilla argentea*	11.7	10.8	0.9	33.2	55.1	0.083	44.9	14.1	12.9	1.2	38.4	47.5	0.093	52.5
*Potentilla canadensis*	12.9	9.6	3.3	47.8	39.3	0.344	60.7	17.3	12.2	5.1	52.9	29.8	0.418	70.2
*Prunus serotina*	23.5	18.1	5.4	50.4	26.1	0.298	73.9	28.6	20	8.6	47.1	24.3	0.430	75.7
*Prunus virginiana*	27	18.1	8.9	54.4	18.6	0.492	81.4	37.7	25.5	12.2	44.7	17.6	0.478	82.4
*Reynoutria japonica*	30.1	22.8	7.3	45.2	24.7	0.320	75.3	30.2	23.1	7.1	45.5	24.3	0.307	75.7

Note

C = percentage of all complete BUSCO matches in the respective database (C = S + D where S = percentage of complete and single‐copy BUSCO matches in the respective database and D = percentage of complete and duplicated BUSCO matches in the respective database); F = percentage of fragmented BUSCO matches in the respective database; M = percentage of missing BUSCO matches in the respective database; D/S = ratio of duplicated to single‐copy complete sequences BUSCO matches in the respective database; C+F = percentage of complete and fragmented BUSCO matches in the respective database.

**FIGURE 5:**
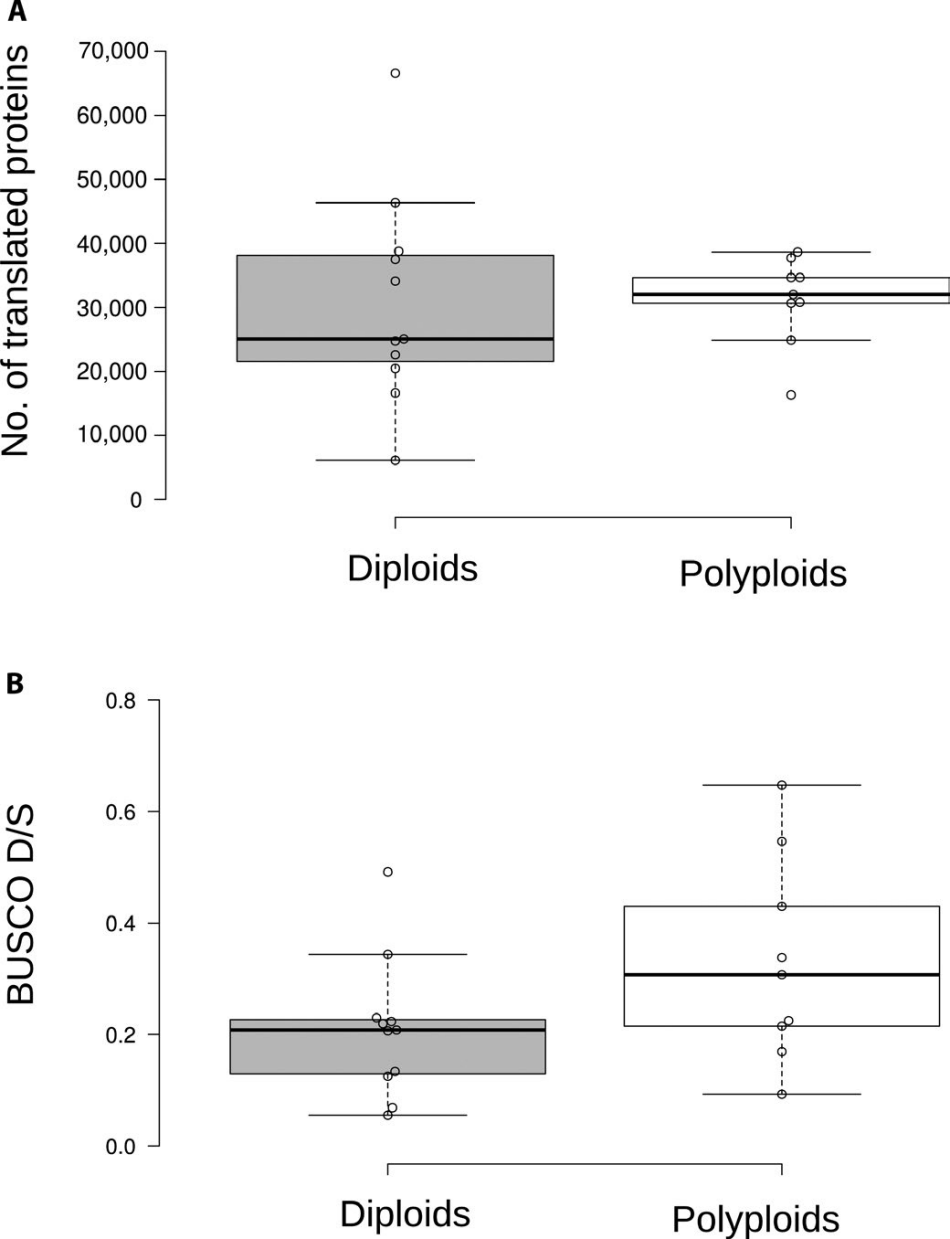
Comparison of (A) the number of translated proteins and (B) BUSCO duplicated/single copy (D/S) ratio for assemblies of diploid and polyploid species. In neither case were diploids significantly different from polyploids.

Polyploid species did not have significantly more translated proteins than diploid species, with 31,152 average proteins translated compared to 30,804 (Fig. 5A; two‐tailed *t*‐test: *P* = 0.95). Similarly, polyploid species did not have a significantly higher proportion of duplicated BUSCO matches than diploids (Fig. 5B; two‐tailed *t*‐test: *P* = 0.11). In some cases, the number of proteins or duplicated BUSCO proportion was lower when comparing a polyploid species with its related diploids (e.g., *Dryopteris* Adans.). This may be due to variation in read and/or assembly quality rather than differences in the biology of these species. However, it is not clear that this is due to differences in data quality because in most cases, including *Dryopteris*, all of the species have similarly high read depth (>20 Gbp).

**FIGURE 6:**
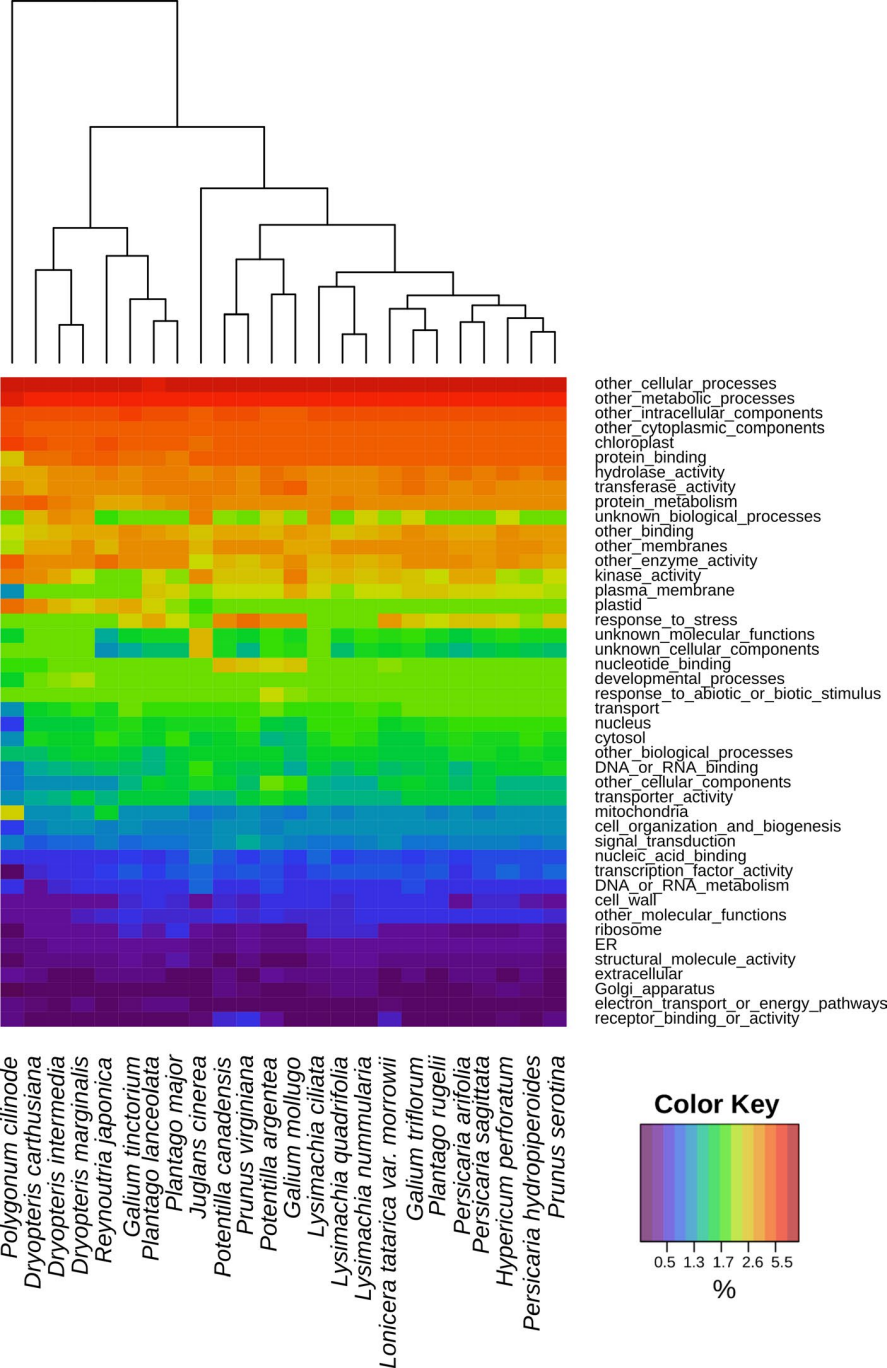
Heat map of Gene Ontology (GO) slim categories present in the entire transcriptome of each species. Each column represents the annotated GO categories from each analyzed transcriptome, whereas the rows represent a particular GO category. The colors of the heat map represent the percentage of the transcriptome represented by a particular GO category, with red being highest and purple lowest. The overall ranking of GO category rows was determined by the ranking of GO annotations in the transcriptome of *Lysimachia ciliata*. Hierarchical clustering was used to organize the heatmap columns.

GO annotations of the transcriptomes of the 24 species were largely similar (Fig. 6). Categories such as *other cellular processes*, *other metabolic processes*, and *other intracellular components* were the largest fraction of all transcriptomes, whereas *receptor binding or activity* and *electron transport or energy pathways* were among the smallest. The rank order of each GO‐slim category was largely consistent across most species. Species from the same genus were sometimes clustered together by the similarity of their GO‐slim representations, such as in *Dryopteris* and *Lysimachia* L., but in most cases the species were not clustered with their congeners. *Polygonum cilinode* Michx. was unique in having many differences in GO category rank compared to the other taxa. It was also the lowest‐scoring transcriptome assembly, with only 6088 translated proteins and nearly 80% of BUSCO genes missing (Table 2).

## DISCUSSION

Overall, the RNA sampling approach we developed and employed (Field Setup 2 of Yang et al., 2017) allowed us to sequence and assemble RNA‐seq data from a diverse range of species at Harvard Forest. The transcriptomes we assembled for 24 species of vascular plants at Harvard Forest appear to be relatively high quality and consistent with our expectations for de novo plant transcriptome assemblies. Our assemblies were reasonably complete, with more than 70% of BUSCO genes present on average. This is a similar distribution of BUSCO scores to those in the recently published 1KP project (Carpenter et al., 2019; One Thousand Plant Transcriptomes Initiative, 2019) and other studies (Blande et al., 2017; Evkaikina et al., 2017; Pokorn et al., 2017; Weisberg et al., 2017). In our analyses, BUSCO scores and scaffold numbers appear to plateau after approximately 20 Gbp of sequencing effort for diploid and polyploid species, but previous studies indicate that reference assemblies of similar quality can be generated with substantially less sequencing effort for high‐quality RNA samples. For example, data from the 1KP project suggest that as little as 2–3 Gbp of read depth is sufficient (Carpenter et al., 2019). Larger amounts of data were collected in this project to facilitate future gene expression analyses. Notably, the few samples that had low BUSCO scores or BUSCO scores that were relatively low for the sequencing effort, such as *Polygonum cilinode*, also had lower numbers of translated proteins but more scaffolds than most species. The relatively poor quality of these outlier assemblies is likely related to lower RNA quality rather than to sequencing effort or ploidal level. In contrast, assemblies with higher BUSCO scores yield translated protein numbers that are more consistent with the number of genes in sequenced plant genomes (Michael, 2014; Wendel et al., 2016). For example, our transcriptomes of *Prunus virginiana* L. and *P. serotina* Ehrh. contained 38,773 and 30,812 translated proteins each. Genomes of related *Prunus* L. species had similar numbers of annotated genes, including 27,852 in *P. persica* (L.) Batsch (International Peach Genome Initiative et al., 2013), 41,294 in *P. yedoensis* Matsum. (Baek et al., 2018), and 43,349 in *P. avium* (L.) L. (Shirasawa et al., 2017). However, these comparisons should be interpreted cautiously because transcriptome assemblies can contain multiple isoforms of protein‐coding genes. Like many transcriptome assemblies (Johnson et al., 2012; Carpenter et al., 2019; Patterson et al., 2019), our assemblies also contain a large number of small scaffolds (<300 bp). Small scaffolds are likely artifacts of library amplification and sequencing, considering that most did not translate to a known plant protein sequence.

We found no significant difference in the number of translated transcripts between the diploid and polyploid transcriptome assemblies. Although this could be due to the modest sample size or variation in the age and fractionation level of polyploids, it may also reflect biological differences in expressed transcriptome size and diversity that impact the number of assembled transcripts. Under a simple null model of polyploid transcriptome size, one may expect to observe an approximate doubling of the diploid transcriptome size that may translate to doubling the number of assembled transcripts. However, recent research indicates polyploid transcriptomes may be smaller than expected. Research in *Glycine* Willd. has found that the expressed transcriptome size of polyploid species is less than 2× the diploid size (Coate and Doyle, 2015; Doyle and Coate, 2018; Visger et al., 2019). For example, the transcriptome of the allotetraploid *G. dolichocarpa* Tateishi & H. Ohashi was 1.4× the size of its diploid progenitors (Coate and Doyle, 2010). The apparent lower‐than‐expected level of the quantity of gene expression in polyploids may be an artifact of comparing diploids and polyploids without accounting for differences in cell numbers or biomass (Coate and Doyle, 2015; Doyle and Coate, 2018; Visger et al., 2019). However, smaller transcriptome sizes in polyploids may also be related to which genes are expressed at a given time or in a particular tissue. This is likely relevant when comparing the assembled gene space for diploid and polyploid transcriptomes, as we do here. Our non‐model reference transcriptomes are built from the expressed genes in each sample rather than being based on a reference genome collection. Thus, only genes and alleles that are expressed will be captured in our assemblies and observed in our comparisons. Not all genes or alleles in a polyploid need to be expressed at one time and the overall diversity of the transcriptome at any given time may look more like a diploid, with other alleles being expressed at different times or tissues. Indeed, differential homoeolog silencing is well characterized in polyploid plants (Adams et al., 2003; Coate and Doyle, 2010) and may reduce the sampled transcript diversity of a polyploid genome. If this is the case, we would expect that sampling across more tissues, development times, and environments would lead to greater sampling of the polyploid gene space. Although RNA spike‐ins and cell counting may improve differential expression analyses (Visger et al., 2019), capturing the full genome diversity of non‐model polyploid species from RNA‐seq assemblies remains an additional challenge.

Our pilot study of RNA‐seq sampling of diverse species in the field demonstrated some familiar challenges. Building on our past experience with extracting RNA from diverse species (Barker et al., 2008; Dempewolf et al., 2010; Der et al., 2011; Lai et al., 2012; Dlugosch et al., 2013; Hodgins et al., 2014; Barker et al., 2016b; Mandáková et al., 2017; Qi et al., 2017; Yang et al., 2017; An et al., 2019; Carpenter et al., 2019), we developed an approach for this study to obtain high‐quality RNA from field samples (Field Setup 2 of Yang et al., 2017). We found that flash‐freezing leaves in liquid nitrogen in situ for later RNA extraction worked well for our diverse samples. A few samples, especially *Polygonum cilinode*, yielded lower‐quality RNA, which could potentially be related to leaf age at the time of sampling. Different RNA extraction methods will be needed to deal with the secondary compounds (e.g., polyphenolics) that are present in mature and senescing tissues. Recovering high‐quality RNA in the field, across a range of time points and from leaves of different ages, will be a challenge for future studies.

Other challenges that will need to be overcome are associated with sampling at NEON sites. Sampling within NEON permanent plots is generally not allowed for collections outside of NEON’s own standard protocol, and therefore our sampling was limited to sites adjacent to NEON plots. This limitation raises some significant issues for researchers who wish to leverage data being collected within NEON sites (https://data.neonscience.org/). First, many NEON sites are located in areas where there is no similar adjacent field site available for sampling, due to land restrictions or ecological variation. We ultimately selected Harvard Forest because we could sample at sites outside of the NEON plot itself. The second major issue is that sampling outside of the NEON plot means that there is no guarantee of continued access to plant populations in the future. There is a great opportunity for ecologists and evolutionary biologists to leverage the wealth of data that NEON is generating for our community. However, access for researchers that wish to conduct RNA and DNA sampling of plants (and other organisms) within NEON sites is an essential issue that requires further development across the network. Sequencing costs will continue to decline over the planned 30‐year life span of NEON, and strategies to accommodate sequencing for plants and other eukaryotes will offer opportunities to greatly expand large‐scale studies at the intersection of ecology and evolution.

## AUTHOR CONTRIBUTIONS

H.E.M., K.M.D., and M.S.B. conceived and designed the experiments. H.E.M. and S.A.J collected the samples, extracted the RNA, and collected the vouchers. H.E.M., E.W., Z.L., K.M.D., and M.S.B. analyzed the data. H.E.M., Z.L., K.M.D., and M.S.B. drafted the manuscript, and all authors approved the final manuscript.

## Data Availability

Raw reads for all samples for 24 species are deposited in the National Center for Biotechnology Information (NCBI) Sequence Read Archive (SRP127805: https://www.ncbi.nlm.nih.gov/sra/SRP127805; BioProject: PRJNA422719). Assembled transcriptomes for each species are archived on Zenodo and available at https://doi.org/10.5281/zenodo.3727312 (Marx et al., 2020).

## APPENDIX 1 Sample collection number, sampling date, time of day, and locality information, with catalog numbers for vouchers deposited at the University of Arizona Herbarium (ARIZ). RIN (RNA integrity number) scores for each sample are also included.

**Table:**

Family	Species	Collection no.	Sampling date	Sampling time	Sampling locality information	Latitude	Longitude	ARIZ catalog no.
Dryopteridaceae	*Dryopteris carthusiana*	2016‐015	16‐Jul‐16	19:51	Harvard Forest, 26 Prospect Hill Rd., Petersham, MA, 0136, USA. Worcester County. Prospect Hill Tract, Compartment P1. Rd. to Lyford House, ~50 m east of State Route 32. Along bank on south side (south facing), in *Acer saccharum* dominated forest. Scattered in leaf litter.	42.52798	−72.18665	435313
Dryopteridaceae	*Dryopteris carthusiana*	2016‐050	16‐Aug‐16	12:19	Harvard Forest, 26 Prospect Hill Rd., Petersham, MA, 0136, USA. Worcester County. Prospect Hill Tract, Compartment P1. South‐facing slope on the roadway to Lyford House.	42.52789	−72.18668	435265
Dryopteridaceae	*Dryopteris intermedia*	2016‐028	17‐Jul‐16	17:30	Harvard Forest, 26 Prospect Hill Rd., Petersham, MA, 0136, USA. Worcester County. Simes Tract, Compartment Simes. Along path north of gate #29 (north of Dugway Rd.).	42.46747	−72.21249	435364
Dryopteridaceae	*Dryopteris intermedia*	2016‐066	17‐Aug‐16	13:01	Harvard Forest, 26 Prospect Hill Rd., Petersham, MA, 0136, USA. Worcester County. Simes Tract, Compartment Simes. Along path north of gate #29 (north of Dugway Rd.).	42.46762	−72.21259	435293
Dryopteridaceae	*Dryopteris marginalis*	2016‐029	17‐Jul‐16	17:30	Harvard Forest, 26 Prospect Hill Rd., Petersham, MA, 0136, USA. Worcester County. Simes Tract, Compartment Simes. Along path north of gate #29 (north of Dugway Rd.).	42.46747	−72.21249	435363
Dryopteridaceae	*Dryopteris marginalis*	2016‐067	17‐Aug‐16	13:06	Harvard Forest, 26 Prospect Hill Rd., Petersham, MA, 0136, USA. Worcester County. Simes Tract, Compartment Simes. Along path north of gate #29 (north of Dugway Rd.).	42.46762	−72.21259	435294
Rubiaceae	*Galium mollugo*	2016‐008	16‐Jul‐16	10:23	Harvard Forest, 26 Prospect Hill Rd., Petersham, MA, 0136, USA. Worcester County. Prospect Hill Tract, Compartment P1. Shed SE of Torrey Lab, along forest edge.	42.5319	−72.18893	435348
Rubiaceae	*Galium mollugo*	2016‐044	15‐Aug‐16	17:31	Harvard Forest, 26 Prospect Hill Rd., Petersham, MA, 0136, USA. Worcester County. Prospect Hill Tract, Compartment P1. Around wood shed and generator, along forest margin east of Torrey Hall. South of dumpster and parking.	42.53189	−72.18892	435259
Rubiaceae	*Galium tinctorium*	2016‐022	17‐Jul‐16	9:40	Harvard Forest, 26 Prospect Hill Rd., Petersham, MA, 0136, USA. Worcester County. Slab City Tract, Compartment S6. Along trail SE of gate #26, to Connor Pond marsh margin. Followed margin back north ~10 m to opening in forest (mixed *Thuja*, *Acer*, *Pinus*). Collected at forest/marsh margin.	42.46639	−72.16238	435357
Rubiaceae	*Galium tinctorium*	2016‐059	16‐Aug‐16	17:37	Harvard Forest, 26 Prospect Hill Rd., Petersham, MA, 0136, USA. Worcester County. Slab City Tract, Compartment S6. Along trail SE of gate #26, to Connor Pond marsh margin. Followed margin back north ~10 m to opening in forest (mixed *Thuja*, *Acer*, *Pinus*). Collected at forest/marsh margin.	42.46631	−72.16244	435288
Rubiaceae	*Galium triflorum*	2016‐030	17‐Jul‐16	17:57	Harvard Forest, 26 Prospect Hill Rd., Petersham, MA, 0136, USA. Worcester County. Simes Tract, Compartment Simes. At gate #26.	42.46616	−72.21214	435379
Rubiaceae	*Galium triflorum*	2016‐069	17‐Aug‐16	13:42	Harvard Forest, 26 Prospect Hill Rd., Petersham, MA, 0136, USA. Worcester County. Simes Tract, Compartment Simes. At gate #26.	42.46608	−72.21198	435296
Hypericaceae	*Hypericum perforatum*	2016‐005	16‐Jul‐16	9:47	Harvard Forest, 26 Prospect Hill Rd., Petersham, MA, 0136, USA. Worcester County. Prospect Hill Tract, Compartment P1. West of gas shed that is SE of Torrey Lab. Sandy parking lot near forest margin.	42.53202	−72.18911	435355
Hypericaceae	*Hypericum perforatum*	2016‐041	15‐Aug‐16	17:20	Harvard Forest, 26 Prospect Hill Rd., Petersham, MA, 0136, USA. Worcester County. Prospect Hill Tract, Compartment P1. West of gas shed that is SE of Torrey Lab. Sandy parking lot near forest margin.	42.53197	−72.18904	435224
Juglandaceae	*Juglans cinerea*	2016‐009	16‐Jul‐16	11:36	Harvard Forest, 26 Prospect Hill Rd., Petersham, MA, 0136, USA. Worcester County. Prospect Hill Tract, Compartment P1. Along south side of trail (Locust Opening Rd.) that heads east from Torrey Lab. ~100 m past gate, next to phone pole (#135‐5).	42.53244	−72.18853	435347
Juglandaceae	*Juglans cinerea*	2016‐045	16‐Aug‐16	10:09	Harvard Forest, 26 Prospect Hill Rd., Petersham, MA, 0136, USA. Worcester County. Prospect Hill Tract, Compartment P1. Along south side of trail (Locust Opening Rd.) that heads east from Torrey Lab. ~100 m past gate, next to phone pole (#135‐5).	42.53265	−72.18901	435260
Caprifoliaceae	*Lonicera tatarica* var. *morrowii*	2016‐001	16‐Jul‐16	9:34	Harvard Forest, 26 Prospect Hill Rd., Petersham, MA, 0136, USA. Worcester County. Prospect Hill Tract, Compartment P1. John H. Torrey Laboratory. North parking lot, along fence margin.	42.54171	−72.18997	435195
Caprifoliaceae	*Lonicera tatarica* var. *morrowii*	2016‐037	15‐Aug‐16	15:12	Harvard Forest, 26 Prospect Hill Rd., Petersham, MA, 0136, USA. Worcester County. Prospect Hill Tract, Compartment P1. John H. Torrey Laboratory. North parking lot, along fence margin.	42.53246	−72.18967	435228
Primulaceae	*Lysimachia ciliata*	2016‐031	17‐Jul‐16	18:01	Harvard Forest, 26 Prospect Hill Rd., Petersham, MA, 0136, USA. Worcester County. Simes Tract, Compartment Simes. At gate #26.	42.46616	−72.21214	435366
Primulaceae	*Lysimachia ciliata*	2016‐070	17‐Aug‐16	13:47	Harvard Forest, 26 Prospect Hill Rd., Petersham, MA, 0136, USA. Worcester County. Simes Tract, Compartment Simes. At gate #26.	42.46608	−72.21198	435297
Primulaceae	*Lysimachia nummularia*	2016‐035	18‐Jul‐16	10:00	Harvard Forest, 26 Prospect Hill Rd., Petersham, MA, 0136, USA. Worcester County. Prospect Hill Tract, Compartment P8. Harvard Farm. East of State Route 32 on Pierce Rd., just west of gate.	42.52539	−72.18459	435351
Primulaceae	*Lysimachia nummularia*	2016‐074	17‐Aug‐16	15:15	Harvard Forest, 26 Prospect Hill Rd., Petersham, MA, 0136, USA. Worcester County. Prospect Hill Tract, Compartment P8. Harvard Farm. East of State Route 32 on Pierce Rd., just west of gate.	42.52341	−72.18465	435299
Primulaceae	*Lysimachia quadrifolia*	2016‐024	17‐Jul‐16	10:50	Harvard Forest, 26 Prospect Hill Rd., Petersham, MA, 0136, USA. Worcester County. Slab City Tract, Compartment S6. East of State Route 122, along trail leading south to gate #26. Forest margin / roadside clearing along fence. Weedy.	42.46713	−72.16381	435359
Primulaceae	*Lysimachia quadrifolia*	2016‐062	17‐Aug‐16	10:20	Harvard Forest, 26 Prospect Hill Rd., Petersham, MA, 0136, USA. Worcester County. Slab City Tract, Compartment S6. East of State Route 122, along trail leading south to gate #26. Forest margin / roadside clearing along fence. Weedy.	42.26717	−72.16374	435290
Polygonaceae	*Persicaria arifolia*	2016‐021	17‐Jul‐16	9:33	Harvard Forest, 26 Prospect Hill Rd., Petersham, MA, 0136, USA. Worcester County. Slab City Tract, Compartment S6. Along trail SE of gate #26, to Connor Pond marsh margin. Followed margin back north ~10 m to opening in forest (mixed *Thuja*, *Acer*, *Pinus*). Collected at forest/marsh margin.	42.46639	−72.16238	435356
Polygonaceae	*Persicaria arifolia*	2016‐058	16‐Aug‐16	17:30	Harvard Forest, 26 Prospect Hill Rd., Petersham, MA, 0136, USA. Worcester County. Slab City Tract, Compartment S6. Along trail SE of gate #26, to Connor Pond marsh margin. Followed margin back north ~10 m to opening in forest (mixed *Thuja*, *Acer*, *Pinus*). Collected at forest/marsh margin.	42.46631	−72.16244	435289
Polygonaceae	*Persicaria hydropiperoides*	2016‐006	16‐Jul‐16	9:50	Harvard Forest, 26 Prospect Hill Rd., Petersham, MA, 0136, USA. Worcester County. Prospect Hill Tract, Compartment P1. West of dumpster SE of Torrey Lab, in sandy lot at forest edge, weedy patch.	42.53198	−72.18908	435350
Polygonaceae	*Persicaria hydropiperoides*	2016‐042	15‐Aug‐16	17:23	Harvard Forest, 26 Prospect Hill Rd., Petersham, MA, 0136, USA. Worcester County. Prospect Hill Tract, Compartment P1. Around dumpster east of Torrey Hall and south of shed.	42.53197	−72.18904	435223
Polygonaceae	*Persicaria sagittata*	2016‐020	17‐Jul‐16	9:31	Harvard Forest, 26 Prospect Hill Rd., Petersham, MA, 0136, USA. Worcester County. Slab City Tract, Compartment S6. Along trail SE of gate #26, to Connor Pond marsh margin. Followed margin back north ~10 m to opening in forest (mixed *Thuja*, *Acer*, *Pinus*).	42.46639	−72.16238	435308
Polygonaceae	*Persicaria sagittata*	2016‐057	16‐Aug‐16	17:24	Harvard Forest, 26 Prospect Hill Rd., Petersham, MA, 0136, USA. Worcester County. Slab City Tract, Compartment S6. Along trail SE of gate #26, to Connor Pond marsh margin. Followed margin back north ~10 m to opening in forest.	42.46631	−72.16244	435257
Plantaginaceae	*Plantago lanceolata*	2016‐018	16‐Jul‐16	20:06	Harvard Forest, 26 Prospect Hill Rd., Petersham, MA, 0136, USA. Worcester County. Prospect Hill Tract, Compartment P1. Lawn west of Lyford House.	42.5282	−72.18615	435310
Plantaginaceae	*Plantago lanceolata*	2016‐053	16‐Aug‐16	13:43	Harvard Forest, 26 Prospect Hill Rd., Petersham, MA, 0136, USA. Worcester County. Prospect Hill Tract, Compartment P1. Lawn west of Lyford House (before fence).	42.52807	−72.18624	435268
Plantaginaceae	*Plantago major*	2016‐003	16‐Jul‐16	9:27	Harvard Forest, 26 Prospect Hill Rd., Petersham, MA, 0136, USA. Worcester County. Prospect Hill Tract, Compartment P1. East of Torrey Lab, in sandy parking lot along forest margin.	42.53218	−72.18916	435354
Plantaginaceae	*Plantago major*	2016‐039	15‐Aug‐16	16:09	Harvard Forest, 26 Prospect Hill Rd., Petersham, MA, 0136, USA. Worcester County. Prospect Hill Tract, Compartment P1. East of Torrey Lab, in sandy parking lot along forest margin.	42.53216	−72.18912	435226
Plantaginaceae	*Plantago rugelii*	2016‐004	16‐Jul‐16	9:30	Harvard Forest, 26 Prospect Hill Rd., Petersham, MA, 0136, USA. Worcester County. Prospect Hill Tract, Compartment P1. East of Torrey Lab, in sandy parking lot along forest margin.	42.53218	‐−72.18916	435352
Plantaginaceae	*Plantago rugelii*	2016‐040	15‐Aug‐16	16:11	Harvard Forest, 26 Prospect Hill Rd., Petersham, MA, 0136, USA. Worcester County. Prospect Hill Tract, Compartment P1. East of Torrey Lab, in sandy parking lot along forest margin.	42.53216	−72.18912	435225
Polygonaceae	*Polygonum cilinode*	2016‐025	17‐Jul‐16	11:13	Harvard Forest, 26 Prospect Hill Rd., Petersham, MA, 0136, USA. Worcester County. Slab City Tract, Compartment S6. West side of State Route 122, just north of gate #27 along guard rail. Disturbed soils.	42.45533	−72.16677	435360
Polygonaceae	*Polygonum cilinode*	2016‐063	17‐Aug‐16	10:47	Harvard Forest, 26 Prospect Hill Rd., Petersham, MA, 0136, USA. Worcester County. Slab City Tract, Compartment S6. West side of State Route 122, just north of gate #27 along guard rail. Disturbed soils.	42.45564	−72.16701	435287
Rosaceae	*Potentilla argentea*	2016‐017	16‐Jul‐16	19:53	Harvard Forest, 26 Prospect Hill Rd., Petersham, MA, 0136, USA. Worcester County. Prospect Hill Tract, Compartment P1. Bordering garage south of Lyford House.	42.52819	−72.18606	435311
Rosaceae	*Potentilla argentea*	2016‐055	16‐Aug‐16	1:51	Harvard Forest, 26 Prospect Hill Rd., Petersham, MA, 0136, USA. Worcester County. Prospect Hill Tract, Compartment P1. In sandy soil of parking area south of Lyford House.	42.52821	−72.18581	435255
Rosaceae	*Potentilla canadensis*	2016‐033	17‐Jul‐16	16:55	Harvard Forest, 26 Prospect Hill Rd., Petersham, MA, 0136, USA. Worcester County. Prospect Hill Tract, Compartment P10. French Rd., east of gate #1. On north side of trail at forest edge, just east of old foundation. With *Acer*, *Fraxinus*, relatively open clearing.	42.53904	−72.19297	435362
Rosaceae	*Potentilla canadensis*	2016‐072	17‐Aug‐16	14:36	Harvard Forest, 26 Prospect Hill Rd., Petersham, MA, 0136, USA. Worcester County. Prospect Hill Tract, Compartment P10. French Road, east of gate #1. On north side of trail at forest edge, just east of old foundation. With *Acer*, *Fraxinus*, relatively open clearing.	42.53907	−72.193	435306
Rosaceae	*Prunus serotina*	2016‐027	17‐Jul‐16	16:38	Harvard Forest, 26 Prospect Hill Rd., Petersham, MA, 0136, USA. Worcester County. Tom Swam Tract, Compartment T1. Sunset Lane, west of State Route 32. About 50 m west of Riley Gate, along road‐turned‐path on left (south) side.	42.49305	−72.19437	435230
Rosaceae	*Prunus serotina*	2016‐065	17‐Aug‐16	12:16	Harvard Forest, 26 Prospect Hill Rd., Petersham, MA, 0136, USA. Worcester County. Tom Swam Tract, Compartment T1. Sunset Lane, west of State Route 32. About 50 m west of Riley Gate, along road‐turned‐path on left (south) side.	42.49274	−72.19431	435292
Rosaceae	*Prunus virginiana*	2016‐026	17‐Jul‐16	15:33	Harvard Forest, 26 Prospect Hill Rd., Petersham, MA, 0136, USA. Worcester County. Tom Swam Tract, Compartment T4. Road heading south from Tom Swamp Road at gate #18 (before gate #20). West side of road.	42.50949	−72.20756	435365
Rosaceae	*Prunus virginiana*	2016‐064	17‐Aug‐16	11:26	Harvard Forest, 26 Prospect Hill Rd., Petersham, MA, 0136, USA. Worcester County. Tom Swam Tract, Compartment T4. Road heading south from Tom Swamp Road at gate #18 (before gate #20). West side of road.	42.5095	−72.20757	435291
Polygonaceae	*Reynoutria japonica*	2016‐034	17‐Jul‐16	19:40	Harvard Forest, 26 Prospect Hill Rd., Petersham, MA, 0136, USA. Worcester County. Prospect Hill Tract, Compartment P10. East side of State Route 32, north of Prospect Hill Rd. Along freeway.	42.53301	−72.1924	435361
Polygonaceae	*Reynoutria japonica*	2016‐075	17‐Aug‐16	16:03	Harvard Forest, 26 Prospect Hill Rd., Petersham, MA, 0136, USA. Worcester County. Prospect Hill Tract, Compartment P10. East side of State Route 32, north of Prospect Hill Rd. Along freeway.	42.53295	−72.19247	435305
